# Regulation of C3a Receptor Signaling in Human Mast Cells by G Protein Coupled Receptor Kinases

**DOI:** 10.1371/journal.pone.0022559

**Published:** 2011-07-25

**Authors:** Qiang Guo, Hariharan Subramanian, Kshitij Gupta, Hydar Ali

**Affiliations:** Department of Pathology, School of Dental Medicine, University of Pennsylvania, Philadelphia, Pennsylvania, United States of America; University of Medicine and Dentistry of New Jersey, United States of America

## Abstract

**Background:**

The complement component C3a activates human mast cells via its cell surface G protein coupled receptor (GPCR) C3aR. For most GPCRs, agonist-induced receptor phosphorylation leads to receptor desensitization, internalization as well as activation of downstream signaling pathways such as ERK1/2 phosphorylation. Previous studies in transfected COS cells overexpressing G protein coupled receptor kinases (GRKs) demonstrated that GRK2, GRK3, GRK5 and GRK6 participate in agonist-induced C3aR phosphorylation. However, the roles of these GRKs on the regulation of C3aR signaling and mediator release in human mast cells remain unknown.

**Methodology/Principal Findings:**

We utilized lentivirus short hairpin (sh)RNA to stably knockdown the expression of GRK2, GRK3, GRK5 and GRK6 in human mast cell lines, HMC-1 and LAD2, that endogenously express C3aR. Silencing GRK2 or GRK3 expression caused a more sustained Ca^2+^ mobilization, attenuated C3aR desensitization, and enhanced degranulation as well as ERK1/2 phosphorylation when compared to shRNA control cells. By contrast, GRK5 or GRK6 knockdown had no effect on C3aR desensitization, but caused a significant decrease in C3a-induced mast cell degranulation. Interestingly, GRK5 or GRK6 knockdown rendered mast cells more responsive to C3a for ERK1/2 phosphorylation.

**Conclusion/Significance:**

This study demonstrates that GRK2 and GRK3 are involved in C3aR desensitization. Furthermore, it reveals the novel finding that GRK5 and GRK6 promote C3a-induced mast cell degranulation but inhibit ERK1/2 phosphorylation via C3aR desensitization-independent mechanisms. These findings thus reveal a new level of complexity for C3aR regulation by GRKs in human mast cells.

## Introduction

The complement component C3a plays an important role in innate immunity and also promotes allergic diseases such as bronchial asthma [1], [2], [3]. G protein coupled receptors for C3a (C3aR) are expressed in human mast cell lines (HMC-1, LAD2), differentiated CD34^+^-derived primary human mast cells as well as skin mast cells [4], [5], [6]. C3a induces Ca^2+^ mobilization, causes substantial degranulation and chemokine generation in human mast cells via the activation of Gi-family of G proteins. Removal of potential phosphorylation sites within the carboxyl terminus of C3aR leads to more robust degranulation when compared to wild-type receptors [7]. These findings are consistent with the idea that, as in many other cell types, receptor phosphorylation desensitizes C3aR expressed in mast cells [8].

Agonist occupied GPCRs are phosphorylated by a family of protein kinases, collectively known as G protein coupled receptor kinases (GRKs). Of the seven known GRKs, four (GRK2, GRK3, GRK5 and GRK6) are expressed ubiquitously. It is well established that GPCR phosphorylation by GRKs leads to the recruitment of β-arrestin, which results in receptor desensitization and internalization [8], [9]. However, the role of specific GRKs on receptor regulation has only been appreciated recently. Studies with siRNA-mediated knockdown of GRKs in HEK293 cells have shown that agonist-induced phosphorylation of angiotensin II type 1A receptor (Gq-coupled) and V2 vasopressin receptors (Gs-coupled) are predominantly mediated by GRK2 and GRK3 [10], [11]. Furthermore, knockdown of these GRKs attenuated both agonist-induced β-arrestin recruitment and receptor desensitization [10], [11].

In addition to desensitization, receptor phosphorylation by GRKs leads to the activation of extracellular signal-regulated kinases (ERK1/2) in a β-arrestin-dependent manner. In HEK293 cells, knockdown of GRK5 and GRK6 inhibits angiotensin II and vasopressin-induced β-arrestin-dependent ERK1/2 phosphorylation [12], [13]. These findings suggest that for angiotensin type IA and vasopressin receptors, agonist-induced receptor phosphorylation by GRK2/GRK3 leads to receptor desensitization but their phosphorylation by GRK5/GRK6 promotes β-arrestin-dependent ERK1/2 phosphorylation. However, for the chemokine receptor CXCR4, GRK2/GRK6 are involved in receptor desensitization whereas GRK3/GRK6 play an important role in positively regulating ERK1/2 activation [14]. In transfected COS cells, overexpression of GRK2, GRK3, GRK5 or GRK6 results in enhancement of agonist-induced C3aR phosphorylation [15]. Our previous studies in a transfected mast cell line, RBL-2H3 indicated that GRK2 may participate in C3aR desensitization [7]. However, the roles of other GRKs on the regulation of receptor function in mast cells remain unknown.

In the present study, we utilized lentivirus shRNA to knockdown the expression of GRK2, GRK3, GRK5 and GRK6 in human mast cells (HMC-1 and LAD2) that endogenously express functional C3aR. Using this system, we report unexpected findings regarding the roles of GRKs on signaling and mediator release in mast cells via both receptor desensitization-dependent and independent pathways.

## Results

### Stable knockdown of GRK2, GRK3, GRK5 and GRK6 expression in the human mast cell line HMC-1 cells

**Figure 1:**
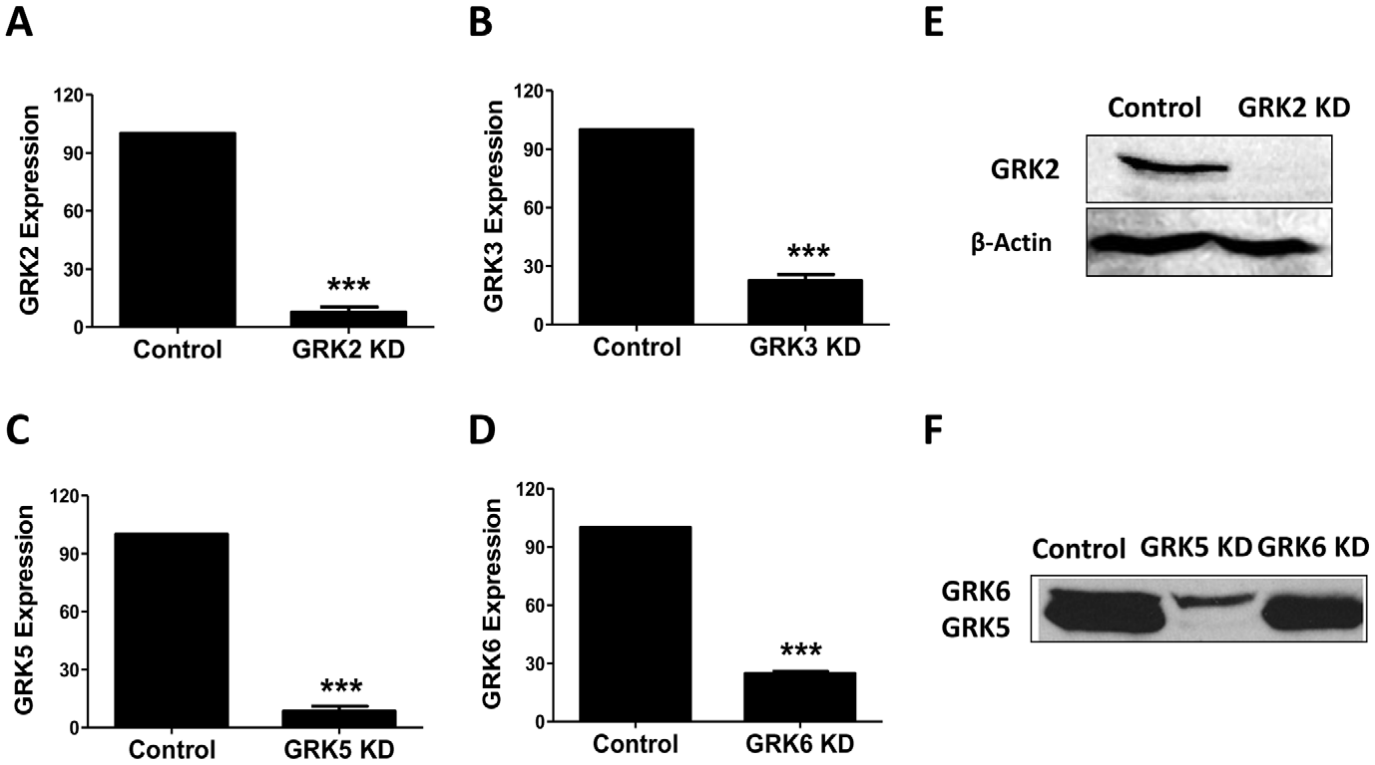
Stable knockdown of GRK2, GRK3, GRK5 and GRK6 in human mast cells. HMC-1 cells were stably transduced with scrambled shRNA control lentivirus (Control) or shRNA lentivirus targeted against GRK2, GRK3, GRK5 and GRK6. (A–D) Quantitative PCR was performed to assess GRK2, GRK3, GRK5 and GRK6 mRNA levels in shRNA control and GRK knockdown (KD) cells. Results are expressed as a ratio of GRK to GAPDH mRNA levels. Data represent the mean ± SEM from three independent experiments. Statistical significance was determined by unpaired two-tailed t test. ***, p<0.001. Representative immunoblots of HMC-1 cells with knockdown of (E) GRK2 and (F) GRK5/GRK6 are shown.

To determine the roles of GRKs on the regulation of C3aR signaling in human mast cells, we used the Mission shRNA lentiviral system to stably knockdown the expression of GRK2, GRK3, GRK5 or GRK6 in a human mast cell line, HMC-1 cells. Cells were separately transduced with 5 different shRNA constructs targeting different regions of each GRK, and the construct that gave the most efficient knockdown was selected for further study. For control, we used a scrambled shRNA construct purchased from Sigma. Although all 5 constructs reduced the expression of each GRK to variable levels, TRCN0000230149 for GRK2, TRCN0000159482 for GRK3, TRCN0000000842 for GRK5 and TRCN0000001368 for GRK6 were the most efficient in knocking down GRKs in HMC-1 cells. These constructs were therefore used for all subsequent studies in HMC-1 and LAD2 mast cells. Using real time quantitative PCR we found that the mRNA levels of GRK2 and GRK5 were reduced by >90% in HMC-1 cells (Fig. 1A and 1C). However, GRK3 and GRK6 mRNA levels were reduced by >80% (Fig. 1B and 1D). Western blot data confirmed almost complete knockdown of GRK2 (Fig. 1E) and GRK5/GRK6 (Fig. 1F). We were not able to detect GRK3 expression with available antibodies.

### GRK2 and GRK3 cause C3aR desensitization in HMC-1 cells but GRK5 and GRK6 do not

**Figure 2:**
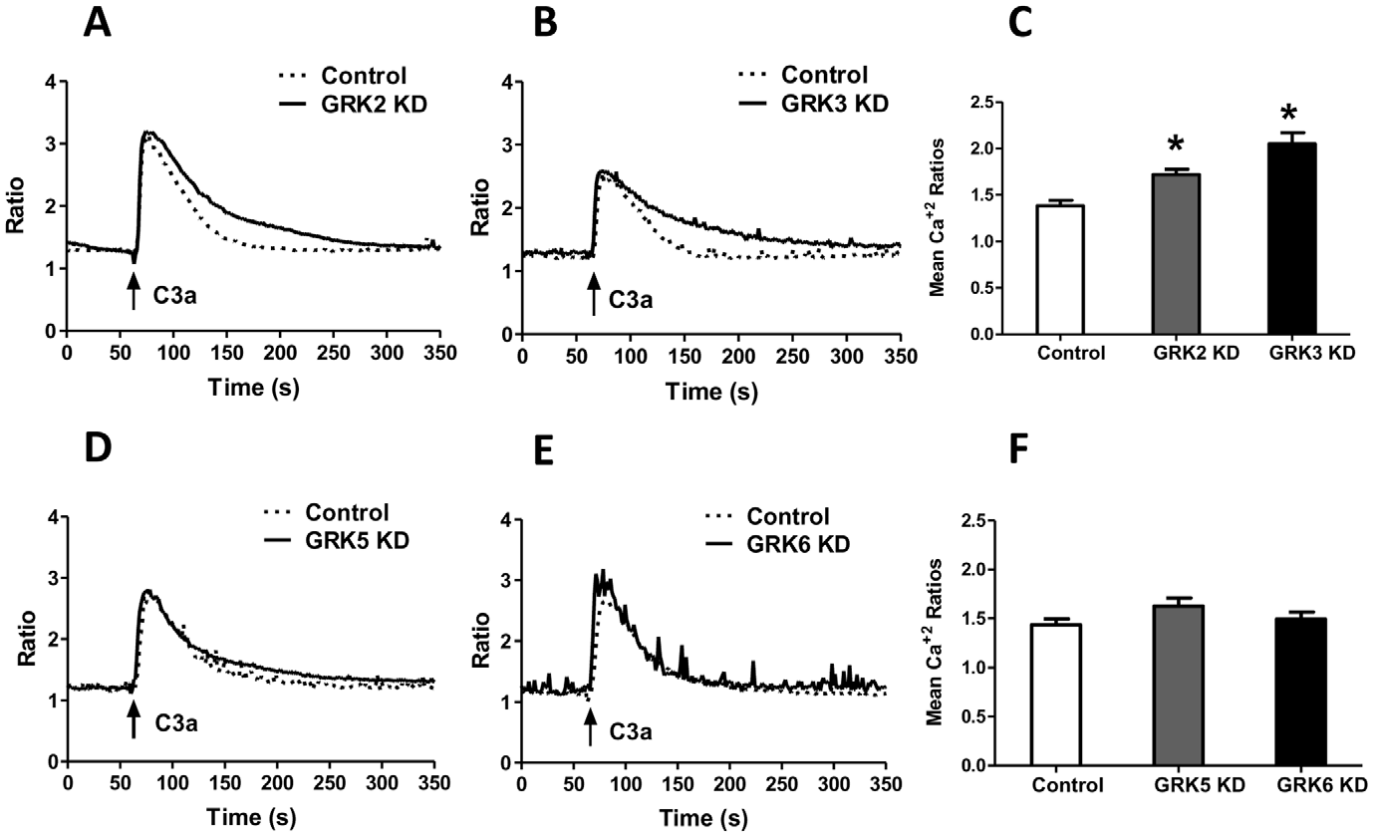
Effects of GRK2/3 and GRK5/GRK6 knockdown on C3a-induced Ca^2+^ mobilization in human mast cells. GRK knockdown (KD) and shRNA control HMC-1 cells were loaded with Indo-1 (1 µM) and Ca^2+^ mobilization in response to C3a (100 nM) was performed as described in the materials and methods. (A) and (B) show Ca^2+^ responses in GRK2 and GRK3 KD cells. (C) shows peak Ca ^2+^ mobilization at 150–200 sec after stimulation. (D) and (E) show Ca ^2+^ responses in GRK5 and GRK6 KD cells. (F) shows peak Ca^2+^ mobilization between 150 and 200 sec after stimulation. For experiments in (A), (B), (D) and (E), representative traces from three independent experiments are shown. For (C) and (F) data represent the mean ± SEM from three independent experiments. Statistical significance was determined by unpaired two-tailed t test. *, p<0.05.

**Figure 3:**
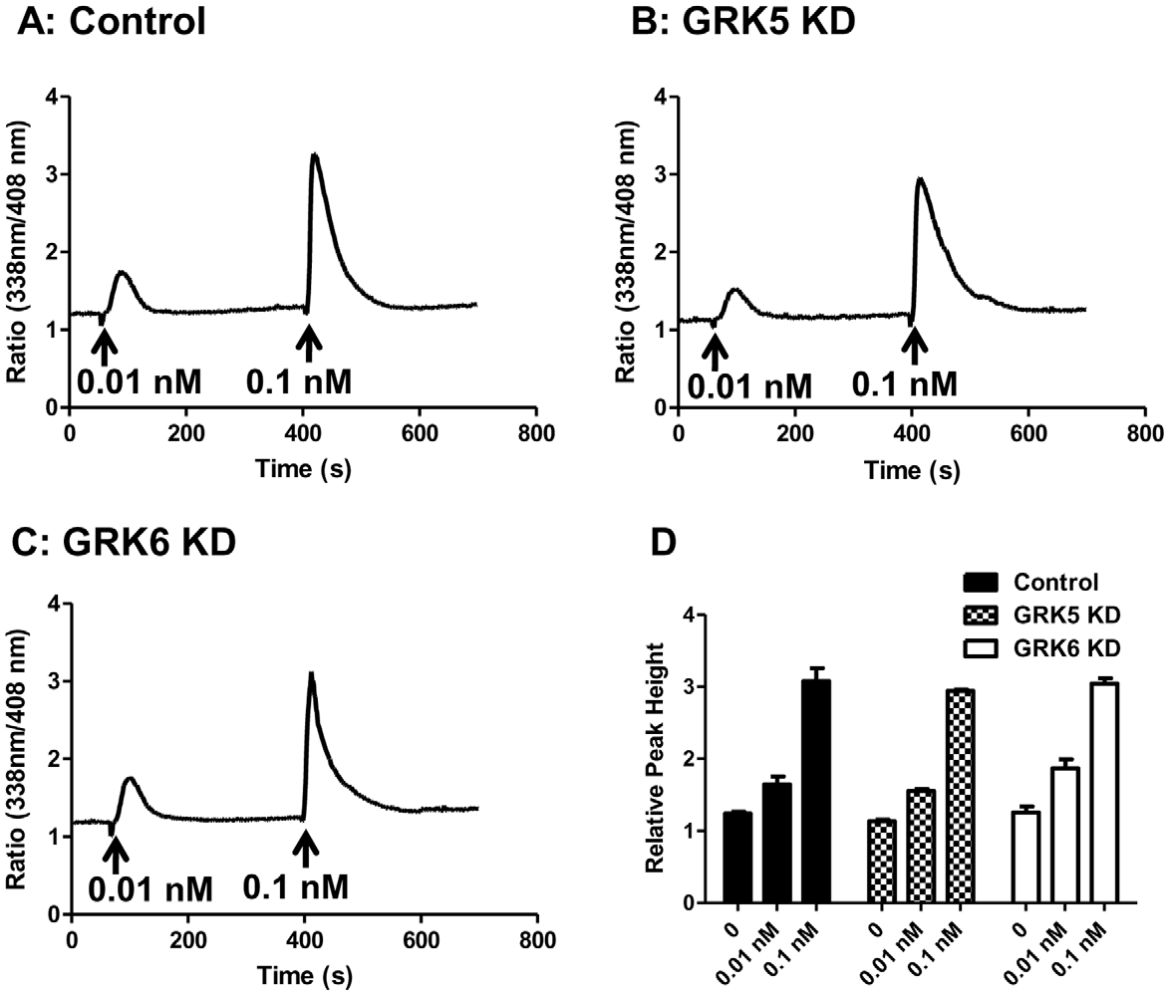
Silencing the expression of GRK5 or GRK6 does not modulate Ca^2+^ mobilization to low concentrations of C3a. (A) shRNA control, (B) GRK5 KD and (C) GRK6 KD HMC-1 cells were loaded with Indo-1 (1 µM), sequentially exposed to C3a 0.01 nM and 0.1 nM C3a and intracellular Ca^2+^ mobilization was determined. Traces for three representative experiments are shown. For (D), peak Ca^2+^ response to C3a in shRNA control, GRK5 and GRK6 KD cells are shown. Data are the mean ± SEM from three independent experiments.

Intracellular Ca^2+^ mobilization provides a rapid, sensitive and real-time assay to measure desensitization [16]. We have previously shown that GPCRs that undergo desensitization respond to agonist with an initial Ca^2+^ spike, which decays rapidly and reaches baseline within ∼3 min [16]. By contrast, phosphorylation-deficient receptors respond to agonist for a similar initial Ca^2+^ spike, which is followed by a sustained response that remains elevated for an extended period of time. We therefore used Ca^2+^ mobilization as an assay to determine the effect of GRK2, GRK3, GRK5, GRK6 silencing on C3aR desensitization. C3a caused a transient Ca^2+^ mobilization in shRNA control cells (Fig. 2). In GRK2 or GRK3 knockdown (KD) cells the initial Ca^2+^ response to C3a (100 nM) was similar to shRNA control cells. However, the response remained elevated for longer time period (Fig. 2; A–C). By contrast, knockdown of GRK5 or GRK6 had no effect on the magnitude or the time course of C3a -induced Ca^2+^ response (Fig. 2; D–F). To rule out the possibility that GRK5 and GRK6 could modulate Ca^2+^ response to C3a at lower concentrations, we determined the effects of silencing these GRKs on responses to 0.01 nM and 0.1 nM C3a. As shown in Fig. 3, absence of these GRKs had no effect on Ca^2+^ responses to C3a even at very low concentrations.

**Figure 4:**
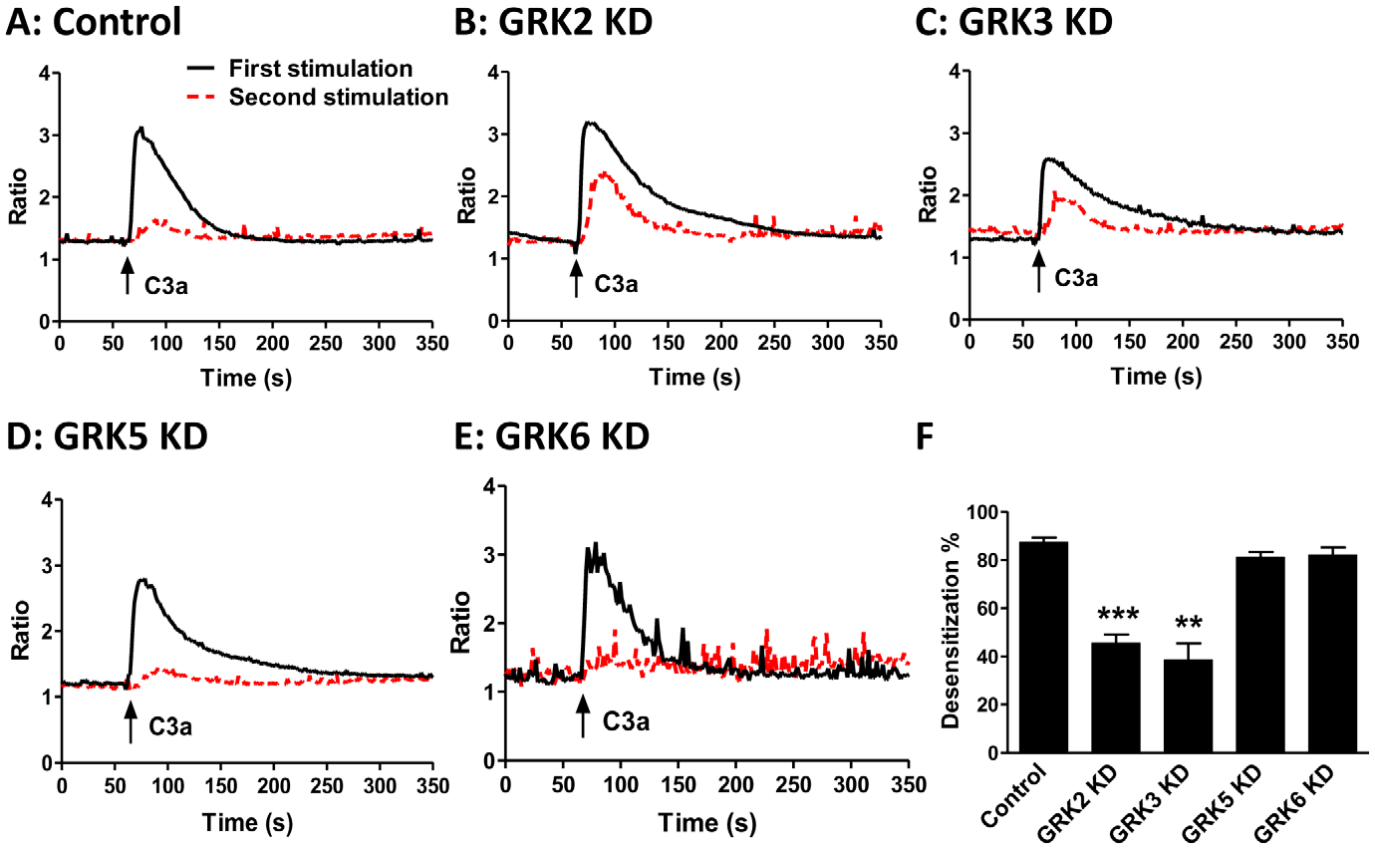
Silencing GRK2 and GRK3 attenuate C3aR desensitization but GRK5 and GRK6 do not. (A) shRNA control, (B) GRK2, (C) GRK3, (D) GRK5 and (E) GRK6 KD cells were loaded with Indo-1 (1 µM), stimulated with C3a (100 nM) for 5 min and intracellular Ca^2+^ mobilization was determined (black solid lines). The cells were washed three times with ice-cold buffer, resuspended in warm buffer and exposed to a second stimulation of C3a (100 nM) and intracellular Ca^2+^ mobilization was again determined (red broken lines). (F) Desensitization was expressed as the percentage decrease in Ca^2+^ response following second stimulation relative to the initial response. Data represent the mean ± SEM from three independent experiments. Statistical significance was determined by unpaired two-tailed t test. **, p<0.01 and ***, p<0.001.

GPCRs that undergo desensitization display reduced responsiveness to a second stimulation with the same agonist [16]. To test further the effects of GRKs on desensitization, shRNA control or GRK KD cells were exposed to C3a and washed 3 times before re-exposure to the same concentration of C3a. shRNA control cells displayed ∼88% reduction in the calcium peak to the second stimulation (Fig. 4A and 4F). By contrast GRK2 and GRK3 KD cells showed ∼45% and ∼40% reduction in the calcium peak to the second stimulation, respectively (Fig. 4; B, C and F). However, Ca^2+^ response to C3a in GRK5 and GRK6 KD cells was very similar to that seen in shRNA control cells (Fig. 4; A, D, E and F). These data demonstrate that while GRK2 and GRK3 participate in C3aR desensitization in HMC-1 cells, GRK5 and GRK6 do not.

### Agonist-induced C3aR internalization does not require GRK2, GRK3, GRK5 or GRK6

**Figure 5:**
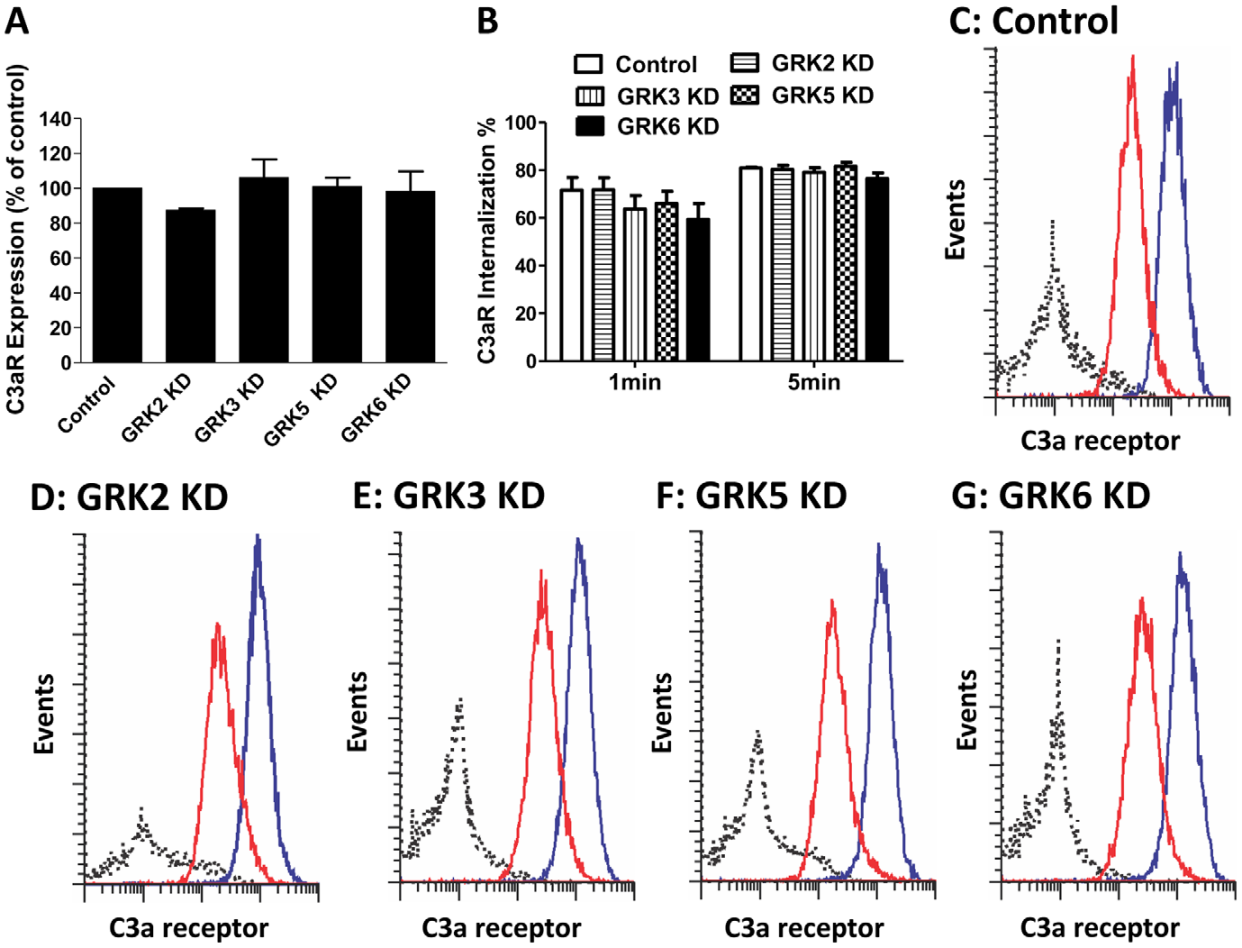
Effects of GRK2/3 and GRK5/6 knockdown on cell surface C3aR expression and agonist-induced receptor internalization. (A) Control shRNA, GRK2, GRK3, GRK5, and GRK6 KD cells were incubated with a mouse anti-C3aR antibody or an isotype control antibody followed by PE-labeled donkey anti-mouse IgG antibody and analyzed by flow cytometry. Cell surface C3aR in each GRK KD cells is shown as a percent of receptor expression in shRNA control cells. (B) shRNA control and cells with knockdown of each GRKs were exposed to buffer or C3a for 1 min or 5 min and cell surface C3aR expression was determined by flow cytometry. Internalization is expressed as percent loss in receptor expression following exposure to C3a. Data represent the mean ± SEM from three independent experiments. (C–G) Representative histogram plots from an internalization experiment following 5 min exposure to buffer (blue line) or C3a (red line) in shRNA control or GRK KD cells are shown.

Receptor internalization is an important mechanism that regulates GPCR signaling. In transfected RBL-2H3 cells, ∼70% of the cell surface receptors undergo internalization following 1 min stimulation with C3a [17]. Furthermore, C3a-induced internalization is blocked by ∼50% in cells expressing a complete phosphorylation-deficient C3aR mutant [17]. This suggests that phosphorylation of C3aR, at least in part, contributes to receptor internalization. Before conducting studies on the role of GRKs on C3aR internalization we performed flow cytometry analysis to determine the impact of GRK knockdown on the cell surface C3aR expression. As shown in Fig. 5A, there was no difference on cell surface expression of C3aR in GRK2, GRK3, GRK5 or GRK6 KD cells when compared to shRNA control cells. To determine the role of GRKs on agonist-induced C3aR internalization, we exposed shRNA control or GRK KD cells with C3a (100 nM) for 1 min and 5 min and determined the extent of cell surface C3aR expression by flow cytometry. Although C3a caused a substantial loss of cell surface receptors in shRNA control cells (∼70%), there was no significant difference in the extent of receptor internalization in GRK2, GRK3, GRK5 or GRK6 KD HMC-1 cells (Fig. 5; B–G). These findings suggest that either GRKs are not involved in agonist-induced C3aR internalization or that multiple GRKs contribute to receptor internalization and loss of one is compensated for by the presence of others.

### C3a-induced ERK1/2 phosphorylation is modulated by GRK2, GRK3, GRK5 and GRK6

**Figure 6:**
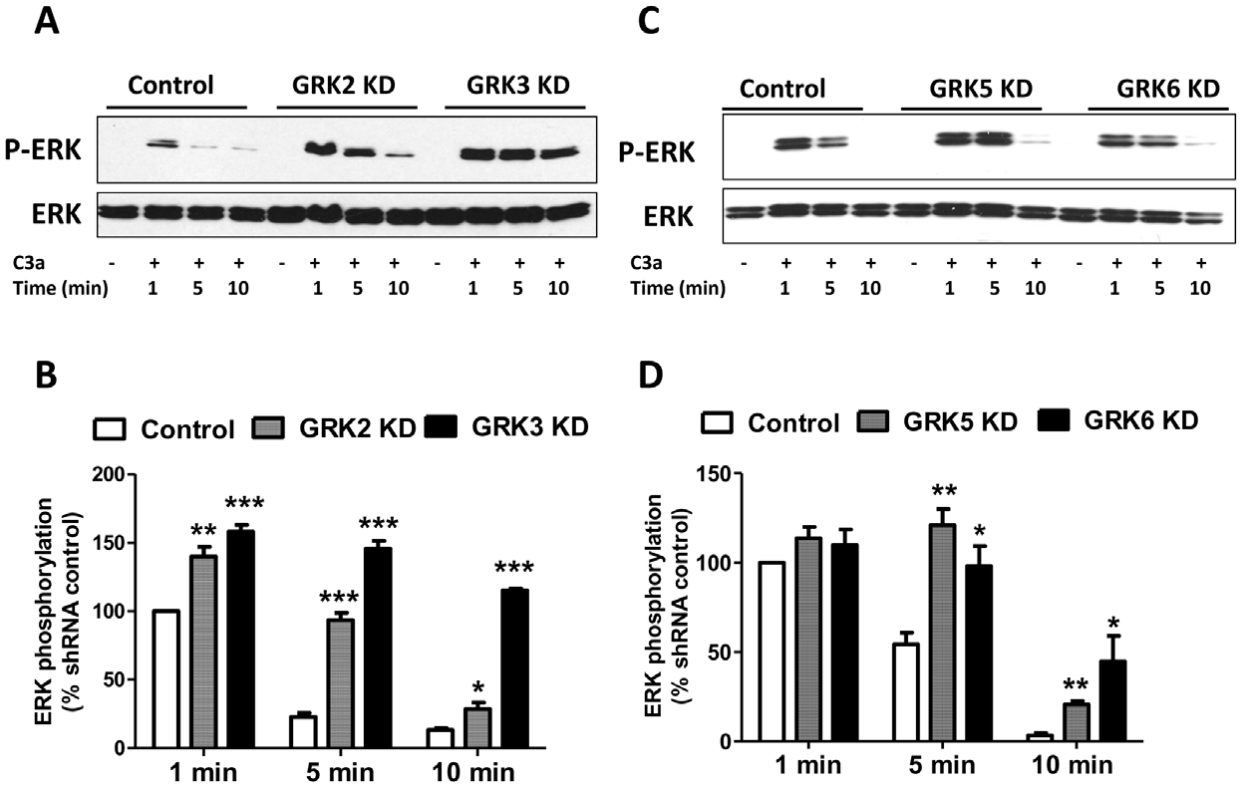
Silencing the expression of GRK2/3 and GRK5/6 enhance C3a-induced ERK1/2 phosphorylation. shRNA control, GRK2, GRK3, GRK5, or GRK6 KD HMC-1 cells were washed with serum-free medium and exposed to C3a (100 nM) for 1, 5 and 10 min. Cell lysates were separated on SDS-PAGE and blots were probed with anti-phospho-ERK1/2 antibody. The blots were then stripped and reprobed with anti-ERK1/2 antibody followed by anti-rabbit IgG-HRP. Immunoreactive band were visualized by SuperSignal West Femto maximum sensitivity substrate. (A, C) Representative immunoblots from three similar experiments are shown. (B, D) ERK1/2 phosphorylation was quantified from 3 experiments using Image J software. Data represent the mean ± SEM from three independent experiments. Statistical significance was determined by unpaired two-tailed t test. *, p<0.05; **, p<0.01 and ***, p<0.001.

**Figure 7:**
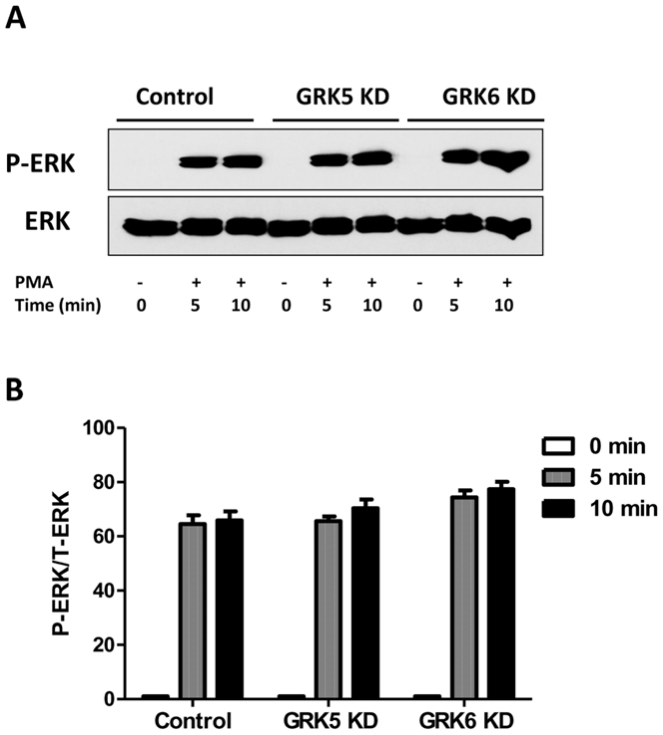
GRKs do not modulate PMA-induced ERK1/2 phosphorylation. shRNA control, GRK5, or GRK6 KD HMC-1 cells were washed with serum-free medium and exposed to phorol-myristate acetate (PMA, 10 nM) for 0, 5 and 10 min. Cell lysates were separated on SDS-PAGE and blots were probed with anti-phospho-ERK1/2 antibody. The blots were then stripped and reprobed with anti-ERK1/2 antibody followed by anti-rabbit IgG-HRP. Immunoreactive band were visualized by SuperSignal West Femto maximum sensitivity substrate. (A) Representative immunoblots from three similar experiments are shown. (B) ERK1/2 phosphorylation from three experiments was quantified using Image J software.

C3a causes transient ERK1/2 phosphorylation in HMC-1 cells [18]. We therefore sought to determine the effects of silencing the expression of GRKs on ERK1/2 phosphorylation in HMC-1 cells. Consistent with our previous studies, we found that C3a causes a transient ERK1/2 phosphorylation that peaked at 1 min and returned to basal levels by 5–10 min. As shown in Fig 6; A and B, silencing GRK2 and GRK3 expression enhanced C3a-induced ERK1/2 phosphorylation at 1, 5 and 10 min. By contrast, GRK5 or GRK6 knockdown had no effect on ERK1/2 phosphorylation at 1 min but rendered the cells responsive to C3a for enhanced ERK1/2 phosphorylation at later time points (5–10 min) (Fig. 6; C and D). To determine the specificity of GRKs for ERK1/2 phosphorylation, we tested the effect of PMA, which bypasses C3aR and activates protein kinase C directly. We found that although PMA (10 nM, at 5 min and 10 min) caused robust ERK1/2 phosphorylation, this response was not significantly altered in GRK5 and GRK6 KD cells (Fig. 7; A and B).

### Distinct roles of GRK2/3 and GRK5/6 on C3a-induced mast cell degranulation

**Figure 8:**
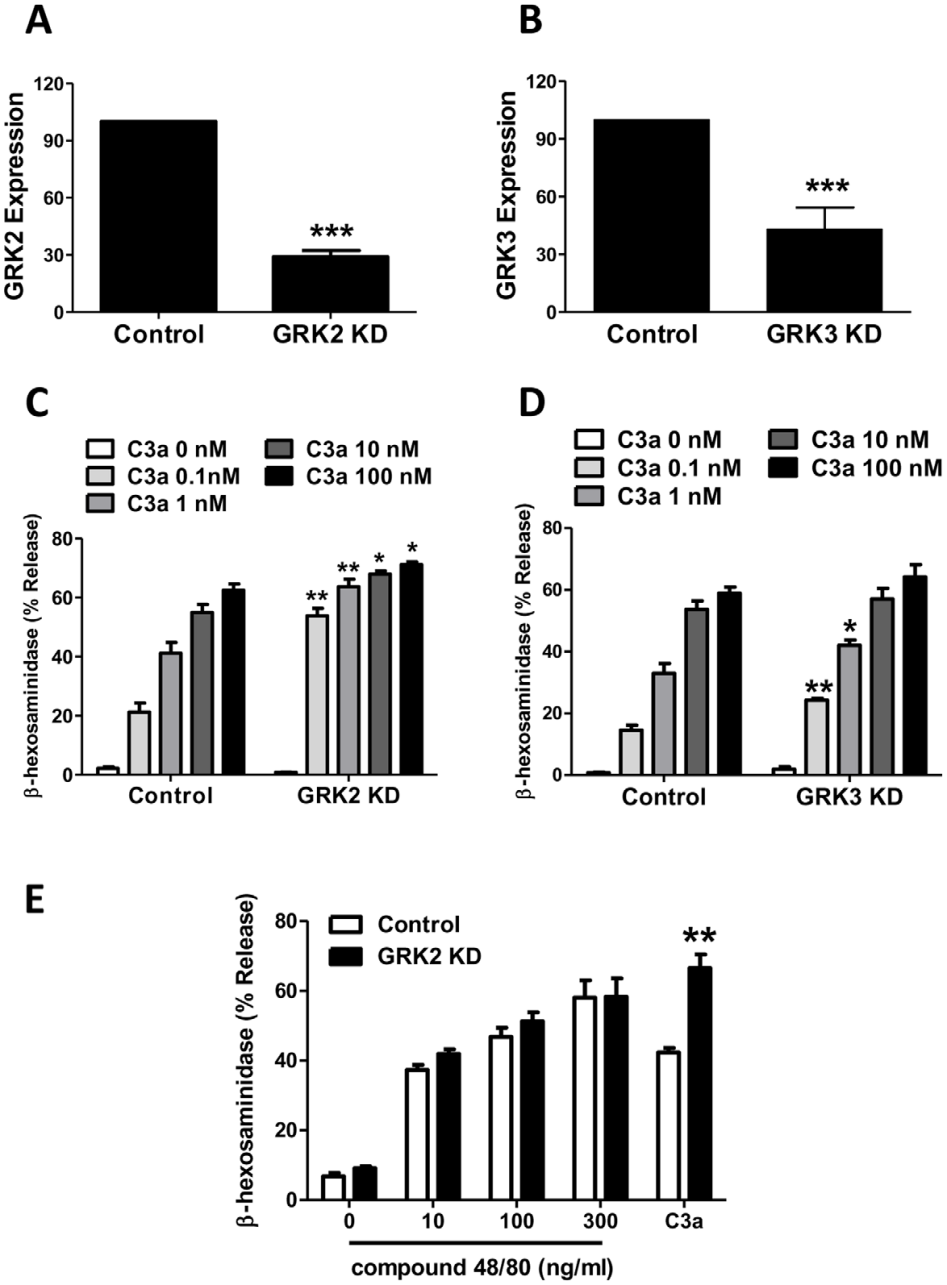
Silencing GRK2 and GRK3 expression enhances C3a-induced mast cell degranulation. LAD2 cells were stably transduced with control shRNA or shRNA lentivirus targeted against GRK2 or GRK3. (A, B) Quantitative PCR was performed to assess the GRK2 and GRK3 knockdown and results are expressed as a ratio of GRK2 or GRK3 to GAPDH mRNA levels. (C, D) Cells were stimulated with different concentrations of C3a (0.1, 1, 10, and 100 nM) and percent degranulation (β-hexosaminidase release) was determined. (E): Control and GRK2 KD cells were exposed to different concentrations of compound 48/80 and percent degranulation was determined. C3a (1 nM) was used as control. Data represent the mean ± SEM from three independent experiments. Statistical significance was determined by unpaired two-tailed t test. *, p<0.05; **, p<0.01; and ***, p<0.001.

Our previous studies in transfected RBL-2H3 cells indicated that GRK2 mediates desensitization of C3aR and inhibits C3a-induced mast cell degranulation [7]. Using shRNA-mediated knockdown of GRKs, we sought to determine the roles of individual GRKs on C3a-induced mast cell degranulation. Because HMC-1 is an immature mast cell line that does not degranulate, we utilized LAD2 mast cells, which endogenously expresses C3aR and responds to C3a for robust degranulation [6]. As GRK2 and GRK3 are involved in C3aR desensitization in HMC-1 cells (Fig. 2 and Fig. 4), we initially focused our studies on these GRKs. We found that although GRK2 mRNA could be reduced by ∼80% (Fig 8A), GRK3 knockdown was less efficient (∼65%) in LAD2 cells (Fig. 8B). Silencing GRK2 or GRK3 resulted in significant enhancement of C3a-induced mast cell degranulation (Fig. 8; C and D). Compound 48/80 induces degranulation in human mast cells via the activation GPCRs, MrgX1 and MrgX2 [19], [20]. To determine the specificity of GRK2 for the regulation of C3a-induced response, we tested the ability of different concentrations of compound 48/80 to induce degranulation in shRNA control and GRK2 KD cells. As shown in Fig. 8E, silencing of GRK2 had no effect on compound 48/80-induced degranulation.

**Figure 9:**
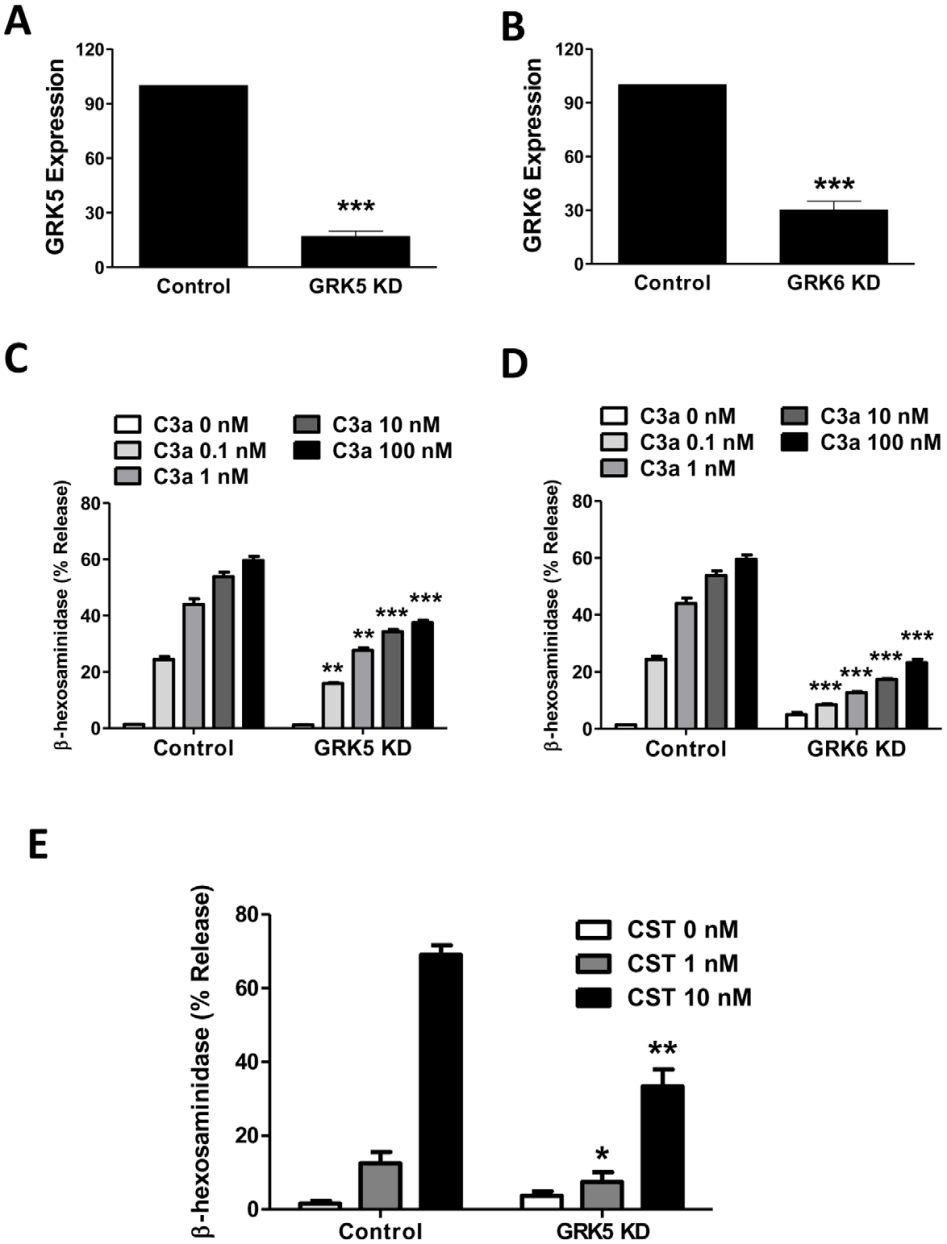
Silencing GRK5 and GRK6 expression inhibits C3a-induced mast cell degranulation. LAD2 cells were stably transduced with control shRNA or shRNA lentivirus targeted against GRK5 or GRK6. (A, B) Quantitative PCR was performed to assess the GRK5 and GRK6 knockdown and results are expressed as a ratio of GRK5 or GRK6 to GAPDH mRNA levels. (C, D) Cells were stimulated with different concentrations of C3a (0.1, 1, 10, and 100 nM) and percent degranulation (β-hexosaminidase release) was determined. (E): shRNA control or GRK5 KD cells were exposed to cortistatin (CST; 1 nM and 10 nM) and percent degranulation was determined. Data represent the mean ± SEM from three independent experiments. Statistical significance was determined by unpaired two-tailed t test. *, p<0.05; **, p<0.01; and ***, p<0.001.

Because GRK5 or GRK6 KD had no effect on C3aR desensitization in HMC-1 cells (Fig. 2, 3, 4), we expected that reduced expression of these GRKs will have little or no impact on C3a-induced mast cell degranulation. To test this possibility, we knocked down the expression of GRK5 and GRK6 in LAD2 mast cells. As for HMC-1 cells (Fig. 1), lentiviral shRNA was efficient in reducing mRNA for these GRKs by ≥75% (Fig. 9; A and B). Unexpectedly, absence of these GRKs caused substantial inhibition of C3a-induced mast cell degranulation (Fig. 9; C and D). To determine the specificity of GRK5 for C3a-induced degranulation, we tested the effect of cortistatin (CST), which activates mast cells via MrgX2 [20], [21]. As for C3a, GRK5 KD significantly reduced CST-induced mast cell degranulation (Fig. 9E).

## Discussion

GRKs are well known for their roles in GPCR desensitization and internalization [8], [9]. They also promote G protein-independent β-arrestin-mediated downstream signaling pathways for ERK1/2 phosphorylation [10], [11], [14]. Most previous studies on GPCR regulation by GRKs have been performed with siRNA-mediated GRK knockdown in HEK293 cells [10], [11], [14]. While tremendous efforts have been devoted towards understanding the mechanisms involved in the regulation of high affinity IgE receptor (FcεRI) almost nothing is known regarding the regulation of GPCR signaling in mast cells. For the present study, we utilized lentivirus shRNA to stably knockdown the expression of GRK2, GRK3, GRK5 and GRK6 in human mast cell lines, HMC-1 and LAD2 that endogenously express C3aR. Using this approach, we have shown that GRK2 and GRK3 cause C3aR desensitization but not receptor internalization. Furthermore, we provide the first demonstration that while GRK5 and GRK6 do not cause C3aR desensitization, they promote C3a-induced mast cell degranulation but inhibit C3a-induced ERK1/2 phosphorylation.

All known GRKs (60–80 kDa) possess a similar structural organization consisting of an amino terminal domain (185 amino acids), a catalytic domain (270 amino acids) and a carboxyl terminal domain (105 to 230 amino acids). There are, however, important differences in the mechanism via which GRK2/GRK3 and GRK5/GRK6 are localized to the proximity of the receptor to induce receptor phosphorylation [8], [22]. GRK2 and GRK3 are found primarily in the cytoplasm and undergo translocation to the plasma membrane upon G protein activation via their interaction with Gβγ subunit and membrane phospholipids. By contrast, GRK5 and GRK6 do not associate with Gβγ but interact with phospholipids or require lipid modification for their association with receptors. We have previously shown that C3a caused enhanced degranulation in RBL-2H3 cells expressing a phosphorylation-deficient C3aR when compared to wild type-C3aR [7]. We also showed that overexpression of GRK2 enhances C3aR phosphorylation to attenuate C3a-induced degranulation [7]. These findings indicated that agonist-induced C3aR phosphorylation by GRK2 promotes C3aR desensitization.

In the present study, we demonstrated that GRK2, GRK3, GRK5 and GRK6 are expressed in two human mast cell lines, HMC-1 and LAD2. Using shRNA-mediated knockdown of GRKs, we confirmed our previous finding regarding the role of GRK2 on agonist-induced C3aR desensitization in mast cells. In addition, we made the novel observation that GRK3 participates in C3aR desensitization in human mast cells. Thus, silencing the expression of GRK2 or GRK3 resulted in more sustained Ca^2+^ mobilization, greater degranulation and enhanced ERK1/2 phosphorylation when compared to shRNA control cells. The carboxyl terminal tail of C3aR possesses nine potential phosphorylation sites and both GRK2 and GRK3 promote agonist-induced C3aR phosphorylation [7], [15]. These findings suggest that in agonist-stimulated mast cells GRK2 and GRK3 may phosphorylate C3aR at the same or distinct sites to promote receptor desensitization.

Previous studies showed that GRK5 and GRK6 promote agonist-induced C3aR phosphorylation in transfected COS cells [15] but the consequence of this phosphorylation remained unknown. An interesting and novel finding of the present study was that silencing GRK5/GRK6 had no effect on the Ca^2+^ response but caused substantial inhibition of C3a-induced mast cell degranulation. This suggests that GRK5 and GRK6 provide stimulatory signals for mast cell degranulation. It is noteworthy that GRK5 phosphorylates β-arrestin-1 [23]. Furthermore, we have recently shown that β-arrestin-1 is required for C3a-induced mast cell degranulation [24]. Previous studies demonstrated that β-arrestin forms a complex with Ral-GDP dissociation stimulator (Ral-GDS) in the cytoplasm of human neutrophils and that activation of fMLP receptor results in the translocation of this complex to the plasma membrane, resulting in the activation of Ral [25]. Phospholipase D (PLD) is an important enzyme that promotes mast cell degranulation [26], [27], [28], [29]. Furthermore, the activity of PLD is regulated by Ral [30], [31]. We have shown that cortistatin, which activates mast cells via MrgX2, also requires the presence of GRK5 for mast cell degranulation. These findings raise the interesting possibility that in response to GPCR activation, GRK5/GRK6-mediated phosphorylation of β-arrestin-1 recruits Ral-GDS-β-arrestin-1 complex to the plasma membrane, resulting in Ral/PLD activation to promote mast cell degranulation. Whether this or other GRK5/GRK6-mediated signaling pathway promotes mast cell degranulation remains to be determined.

It is generally accepted that GRK5 and GRK6 promote GPCR-mediated mitogen-activated protein kinase signaling pathways. Thus, siRNA-mediated knockdown of these GRKs inhibit agonist-induced ERK1/2 phosphorylation [10], [11]. A surprising observation of the present study was that silencing the expression of either GRK5 or GRK6 resulted in enhanced ERK1/2 phosphorylation in response to C3a. The possibility that this enhanced response reflects attenuated C3aR desensitization is unlikely because reduced expression of these GRKs had no effect on C3aR desensitization or internalization. These findings suggest that GRK2/GRK3 and GRK5/GRK6 inhibit C3a-induced ERK1/2 phosphorylation via distinct pathways; one involving receptor desensitization and the other independent of receptor desensitization.

The mechanism by which GRK5/GRK6 inhibit C3a-induced ERK1/2 phosphorylation is not known. Similar to our finding, Barthet et al., [23] recently showed that GRK5 inhibits 5-HT_4_ receptor-mediated ERK1/2 phosphorylation. They also demonstrated that GRK5, but not GRK2, phosphorylates β-arrestin-1 (at Ser^412^) and that this phosphorylation is required for the inhibition of ERK1/2 activity. We have recently shown that silencing the expression of β-arrestin-1 enhances C3a-induced ERK1/2 phosphorylation via a receptor desensitization-independent pathway [24]. This raises the interesting possibility that, as for 5-HT_4_ receptor, agonist-induced C3aR phosphorylation by GRK5/6 recruits β-arrestin-1 to inhibit C3a-induced ERK1/2 phosphorylation. Tipping et al., [32] recently showed that the single β-arrestin present in *Drosophila*, Kurtz (Krz) directly binds to and sequesters an inactive form of ERK, thus preventing its activation by the upstream kinase, MEK. It is therefore possible that C3a induces β-arrestin-1 phosphorylation via GRK5/GRK6 to promote a complex formation between ERK and β-arrestin-1, leading to the inhibition of ERK1/2 activity. Thus, silencing the expression of GRK5/GRK6 or β-arrestin-1 removes this inhibitory constraint to enhance ERK1/2 phosphorylation. Whether this or other mechanisms participate in the regulation of ERK1/2 activity by GRK5/GRK6 in C3a-activated mast cells remains to be determined.

In summary, the present study demonstrates that GRK2 and GRK3 participate in C3aR desensitization in human mast cells. It also provides the novel finding that GRK5 and GRK6 promote C3a-induced mast cell degranulation but inhibit ERK1/2 phosphorylation via mechanisms that are independent of receptor desensitization.

## Materials and Methods

### Materials

Mission shRNA bacterial glycerol stocks for GRK subtypes were purchased from Sigma Life Sciences (St. Louis, MO). Indo-1 AM was from Molecular Probes (Eugene, OR). All tissue culture reagents were purchased from Invitrogen (Gaithersburg, MD). Anti-GRK 2/3 and anti-GRK5/6 was obtained from Millipore (Billerica, MA). Anti-human C3aR was obtained from Santa Cruz Biotechnology (Santa Cruz, CA). Phycoerythrin (PE)-labeled donkey anti-mouse IgG was purchased from eBioscience (San Diego, CA). All recombinant human cytokines were purchased from Peprotech (Rocky Hill, NJ). Rabbit anti-ERK1/2 and anti-phospho-ERK1/2 antibodies were purchased from Cell Signaling (Beverly, MA). LightCycler FastStart RNA Master SYBR Green I was obtained from Roche (Indianapolis, IN). SuperSignal® West Femto Maximum Sensitivity Substrate and HRP-labeled goat anti-rabbit IgG were from Thermo Scientific (Rockford, IL). Purified C3a and Phorbol-12 myristate 13-acetate (PMA) were obtained from Advanced Research Technologies (San Diego, CA) and Calbiochem, (Germany), respectively.

### Mast cell culture

HMC-1 cells were cultured in Iscove's modified Dulbecoo's medium (IMDM) supplemented with 10% FCS, glutamine (2 mM), penicillin (100 IU/mL) and streptomycin (100 µg/mL) [33]. LAD2 cells were maintained in complete StemPro-34 medium supplemented with 100 ng/mL rhSCF [34].

### Lentivirus and stable transduction of shRNAs in mast cells

Lentivirus generation was performed according to the manufacture's manual (Sigma). Cell transduction was conducted by mixing 1.5 ml of virus with 3.5 ml of HMC-1 or LAD2 cell (5×10^6^ cells). Eight hours after transduction, the medium was changed and cells were replenished with fresh medium. After a recovery period of 24 h, puromycin (2 µg/ml) was added to select cells with stable virus integration into the genome. Cells were analyzed for GRK knockdown after one week of antibiotic selection.

### Real-Time PCR

Total RNA was extracted from 1×10^6^ of cells using TRIZOL, treated with DNase I and purified with RNeasy mini Kit according to the manufacture's instruction. Real time PCR was performed with LightCycler FastStart RNA Master SYBR Green I (Roche Applied Science) on a Roche LightCycler2.0 instrument. The primers used for real time PCR were: hGAPDH Forward, 5′-GAGTCCACTGGCGTCTTCA-3′ and hGAPDH Reverse, 5′-GGGGTGCTAAGCAGTTGGT -3′ were used for GAPDH. hGRK2 Forward, 5′-ACT TCAGCGTGCATCGCAT-3′ and hGRK2 Reverse, 5′- GCTTTTTGTCCAGGCACTTCAT-3′ were used for GRK2. hGRK3 Forward, 5′-AGCTGTACCTCAGGTGAAGTT-3′ and hGRK3 Reverse, 5′-AGCTTGCTTTGAGAAAGGATGT-3′ were used for GRK3. hGRK5 Forward, 5′-GACCACACAGACGACGACTTC-3′ and hGRK5 Reverse, 5′-CGTTCAGCTCCTTAAAGCATTC-3′ were used for GRK5. hGRK6 Forward, 5′-TAGCGAACACGGTGCTACTC-3′ and hGRK6 Reverse, 5′-GCTGATGTGAGGGAACTGGA-3′ were used for GRK6. The amplification conditions were as follows;, Reverse transcription at 55°C for 10 min. Denaturation at 95°C for 30 s, 40 cycles at 95°C for 10 s, 66°C (69°C for GRK6) for 10 s, and 72°C for 20 s. Melting curve: conditions were; 95°C, 65°C for 15 s, and 95°C (slope 0.1°C/s). Analysis was performed according to ΔΔCt (delta delta Ct) method. Results are expressed as a ratio of GRK to GAPDH.

### C3a Receptor desensitization assay

Receptor desensitization assay based on Ca^2+^ mobilization was determined as described previously [35]. Briefly, HMC-1 cells (1×10^6^) were washed twice with HEPES buffer (119 mM NaCl, 5 mM KCl, 25 mM HEPES, 5.6 mM Glucose, 0.4 mM MgCl_2_, 1 mM CaCl_2_) containing 1 mg/ml BSA and incubated with 1 µM of Indo-1 for 30 min in dark. Cells were then washed and resuspended in 1.5 ml of the same buffer. The cells were stimulated with 100 nM C3a for 5 min and mobilization of intracellular Ca^2+^ was monitored with a Hitachi F-2500 fluorospectrophotometer. For desensitization assays, cells were removed from the cuvette, washed three times in cold buffer, resuspended in warm buffer and Ca^2+^ mobilization to a subsequent exposure of C3a (100 nM) was determined. Desensitization was expressed as percentage decrease in calcium response following second stimulation relative to the first one.

### Receptor internalization assay based on flow cytometry

Cells were washed and resuspended in fresh medium at a concentration of 0.5 × 10^6^/ml and stimulated with C3a (100 nM) for 1 min or 5 min at 37°C. Cells were washed twice and resuspended in 48 µl of ice-cold FACS buffer (PBS containing 2% FBS). C3aR antibody (2 µL) or Isotype control was added and the cells were incubated on ice for 1 h. After washing twice with cold FACS buffer, cells were resuspended in 48.5 µl of ice-cold FACS buffer. PE-labeled donkey anti-mouse antibody (1.5 µL) was added and the cells were incubated on ice for 1 h. Cells were washed twice and fixed in 300 µl of 2% formaldehyde solution. The samples were acquired and analyzed using FACS Calibur flow cytometer (BD Biosciences).

### ERK1/2 Phosphorylation

shRNA control and GRK knockdown HMC-1 cells were washed twice and resuspended in serum-free IMDM at a concentration of 1×10^6^/ml. Cells were stimulated with C3a (100 nM) or PMA (10 nM) for different time points. Three-fold volume of ice-cold PBS containing 1 mM sodium orthovanadate was added to stop the reaction. Total cell lysates were prepared in RIPA buffer (150 mM NaCl, 1.0% NP-40, 0.5% Sodium-deoxycholate, 0.10% SDS, 50 mM Tris (pH 8.0), 5 mM EDTA, 10 mM NaF, 10 mM Na-pyrophosphate and protease inhibitor cocktail) and subsequently analyzed by Western blot using rabbit polyclonal antibodies for phospho-p44/42 MAPK (pERK1/2) and p44/42 MAPK (ERK1/2).

### Degranulation Assay

LAD2 cells (1.0×10^4^) were seeded into 96-well plates in a total volume of 50 µl of HEPES buffer containing 1 mg/ml BSA and exposed to different concentrations of C3a (0.1, 1, 10 and 100 nM). For total β-hexosaminidase release, control cells were lysed in 50 µl of 0.1% Triton X-100. Aliquots (20 µl) of supernatants or cell lysates were incubated with 20 µl of 1 mM p-nitrophenyl-N-acetyl-β-D-glucosamine for 1.5 h at 37°C. The reaction was stopped by adding 250 µl of a 0.1 M Na_2_CO_3_/0.1 M NaHCO_3_ buffer and absorbance was measured at 405 nm [35].

### Data analysis

The results are expressed as mean ± S.E.M for the values obtained from multiple experiments. Statistical significance was determined by unpaired two-tailed t test. *, p<0.05; **, p<0.01; ***, p<0.001.
